# Supplementary material for: Stream baseline conditions shape functional responses to wastewater: evidence from insect-dominated sites
Source: PeerJ. 2025 Oct 21;13:e20193. doi: 10.7717/peerj.20193 (PMC12551661; doi:10.7717/peerj.20193)
Supplement: Supplemental Information 1 [file peerj-13-20193-s001.docx]

Supplement

2.1S | Site and subset characterisation

A brief summary of communities for the complete and the EPT dominated site datasets is provided in Table S1. In the complete dataset, the total abundance and richness is similar in both the upstream and downstream sites. The EPT abundance was generally higher in the upstream sites, while the EPT richness was comparable for both upstream and downstream sites. However, in the EPT dominated dataset, total abundance was higher in downstream sites compared to upstream sites, while total richness was slightly higher in the upstream sites. Both the EPT abundance and the EPT richness were higher in upstream sites.

**Table S1.** Summary table of upstream and downstream communities for the complete and the least impacted sites dataset. The numbers represent mean values ± SD.

|  | **Complete dataset (n = 338)** | | **EPT dominated site subset (n = 60)** | |
| --- | --- | --- | --- | --- |
|  | Upstream | Downstream | Upstream | Downstream |
| Total abundance | 1,850 ± 2,275 | 1,880 ± 2,071 | 2,058 ± 2,072 | 2,187 ± 2,266 |
| Total richness | 32.0 ± 13.0 | 31.4 ± 12.4 | 37.5 ± 13.6 | 33.5 ± 14.1 |
| EPT abundance | 552 ± 842 | 519 ± 779 | 1,309 ± 1,308 | 1,149 ± 1,383 |
| EPT richness | 13.0 ± 8.0 | 11.7 ± 7.7 | 17.5 ± 8.1 | 14.6 ± 8.1 |

The most prevalent and abundant taxa in the complete dataset were taxa belonging to Gammaridae – *Gammarus roeselii* (Gervais, 1835) and *G. pulex* (Linnaeus, 1758) – Chironomidae, Naididae, Tubificidae, and Baetidae, specifically *Baetis rhodani* (Pictet, 1843). At EPT dominated sites, *G. roeselii*, Chironomidae, as well as *B. rhodani* and *Seratella ignita* (Poda, 1761) dominated in terms of abundance and prevalence. All traits with strong ties to most of these taxa show strong changes when our data were subset by EPT dominance (Fig. S3).


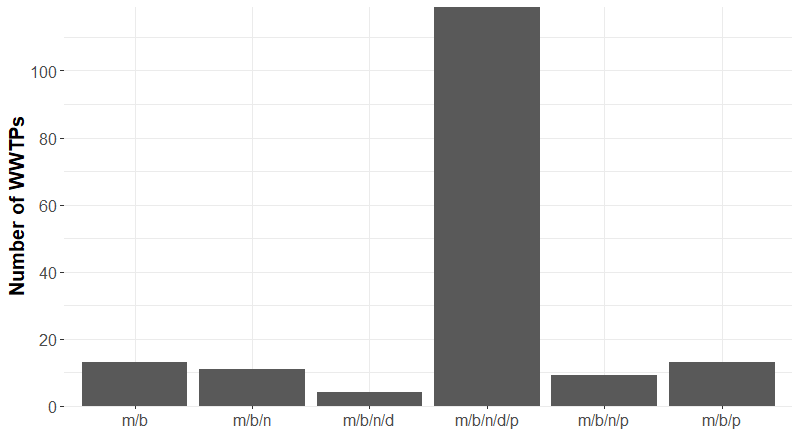


**Fig. S1:** Number of analyzed WWTPs utilizing different treatment methods. m = mechanical; b = biological; n = nitrification; d = denitrification; p = phosphate percipitation.


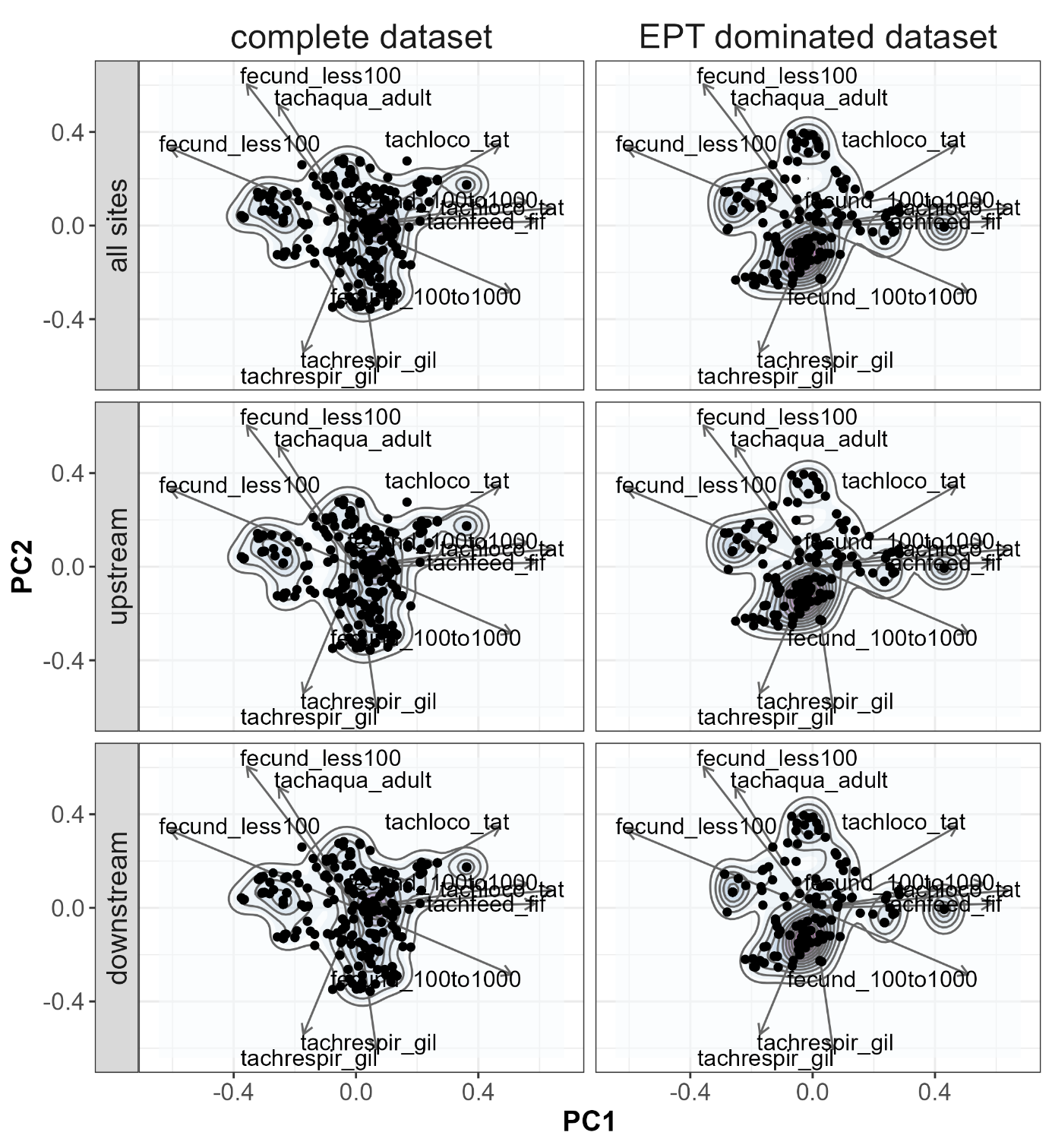


**Fig. S2:** Kernel densities of taxa in functional space. Arrows represent trait loading with a r-square higher than 0.5.


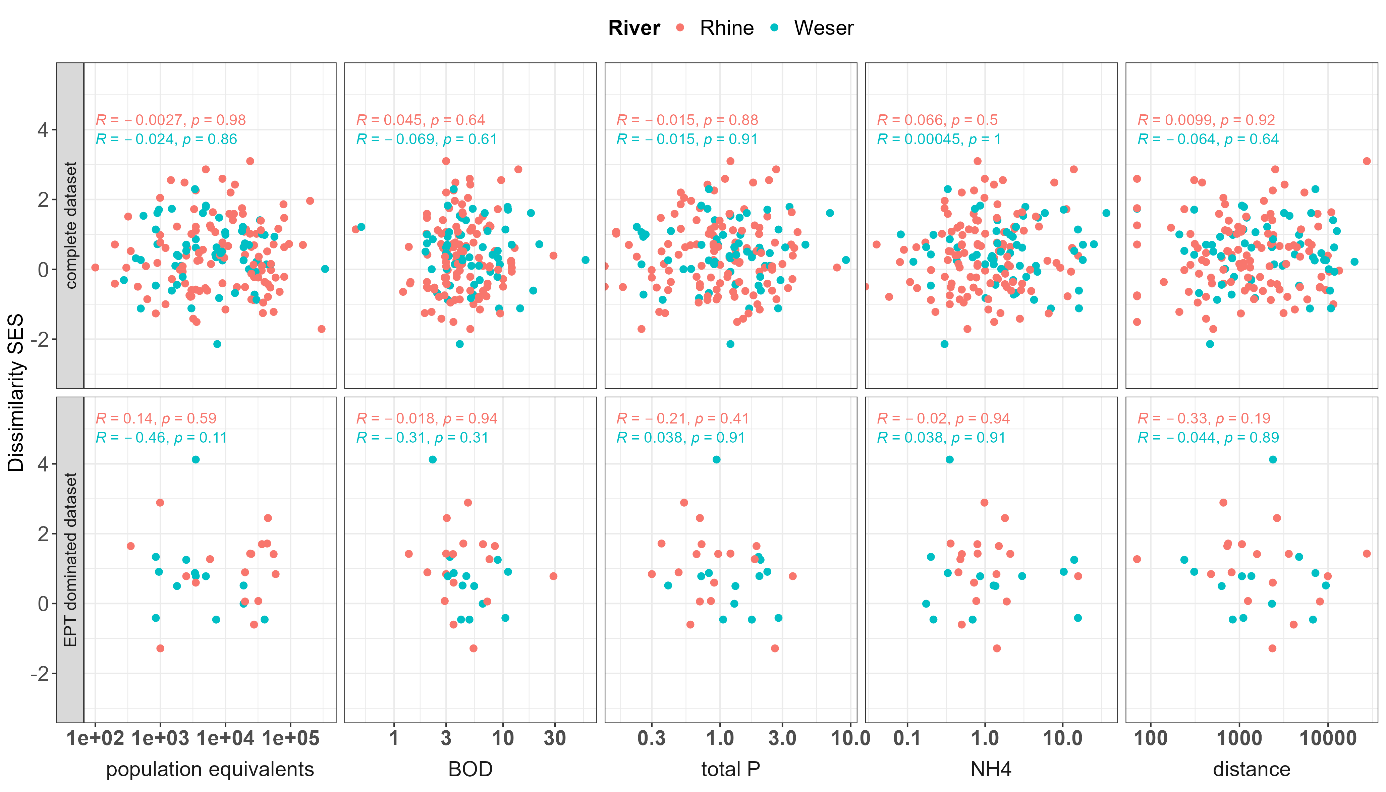


**Fig. S3:** Spearman correlation between standardized effect sizes of functional dissimilarity and population equivalents, instream distance to downstream site and log-transformed WWTP parameters. Points are separated by the two major river systems (Rhine and Weser) of the study region.

| **Size class** | **Population equivalents** |
| --- | --- |
| 1 | <1,000 |
| 2 | 1,000 – 5,000 |
| 3 | 5,001 – 10,000 |
| 4 | 10,001 – 100,000 |
| 5 | >100,000 |

**Table S2:** Size classes and population equivalents for German WWTPs


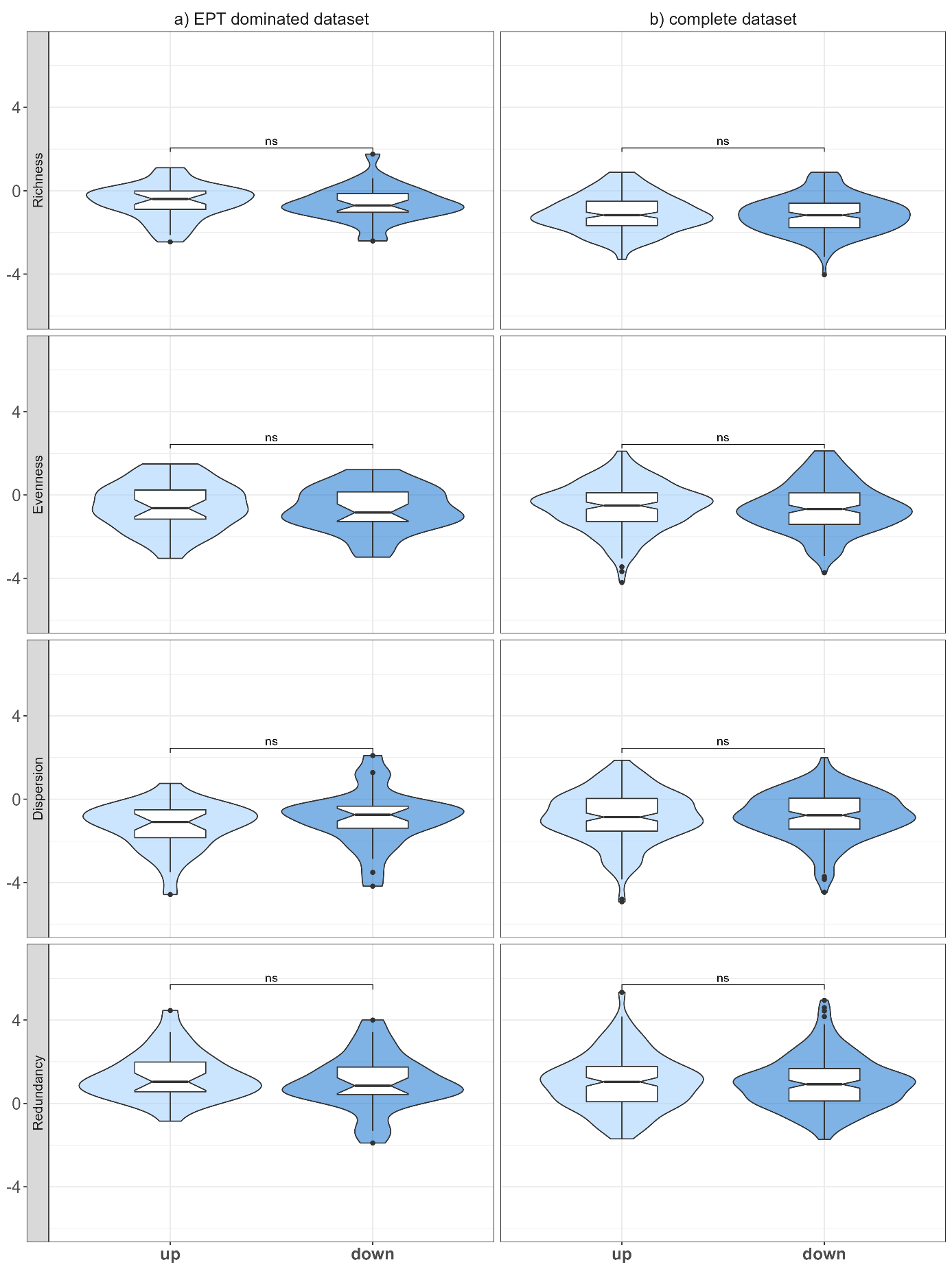


**Fig. S4:** Median standardised effect sizes of functional alpha diversity metrices for the EPT dominant subset and the complete dataset. Significant changes (p < 0.05; paired Wilcoxon test) are indicated by asterisks, while non-significant comparisons are indicated by ns.

**Table S3:** List of analyzed WWTPs and their respective streams

| **UTM-East** | **UTM-North** | **WWTP ID** | **WWTP name** | **stream name** |  |
| --- | --- | --- | --- | --- | --- |
| 460067 | 5616626 | 06532002010-1 | Bischoffen | Aar |  |
| 437752 | 5554179 | 06439015010-1 | Taunusstein / Bleidenstadt | Aar |  |
| 530775 | 5690583 | 06633001010-1 | Ahnatal / Heckershausen | Ahne |  |
| 479803 | 5624255 | 06534014332-1 | Marburg / Haddamshausen | Allna |  |
| 525365 | 5601757 | 06535008020-1 | Herbstein | Alte Hasel |  |
| 528021 | 5602865 | 06535008040-1 | Herbstein / Rixfeld | Alte Hasel |  |
| 512596 | 5631118 | 06535002010-1 | Antrifttal / Bernsburg | Antreff |  |
| 477082 | 5642338 | 06534015020-1 | Münchhausen / Niederasphe | Asphe |  |
| 440316 | 5614302 | 06532004050-1 | Breitscheid / Rabenscheid | Aubach |  |
| 485772 | 5591668 | 06531011070-1 | Lich / Muschenheim | Augraben |  |
| 523449 | 5613599 | 06535011090-1 | Lauterbach (Hessen) / Wallenrod | Bach von Wallenrod |  |
| 491975 | 5645814 | 06635017020-1 | Rosenthal | Bentreff |  |
| 519865 | 5559051 | 06435003010-2 | Biebergemünd / Lanzingen | Bieber |  |
| 472611 | 5606388 | 06531002050-1 | Biebertal / Rodheim-Bieber | Bieber |  |
| 486029 | 5545950 | 06438005010-1 | Heusenstamm | Bieber |  |
| 518982 | 5576440 | 06440013030-1 | Kefenrod / Helfersdorf | Bracht |  |
| 441265 | 5589155 | 06533001040-1 | Beselich / Schupbach | Brandbach |  |
| 521714 | 5606419 | 06535012010-1 | Lautertal (Vogelsberg) / Dirlammen | Brenderwasser |  |
| 452673 | 5555593 | 06439011030-1 | Niedernhausen | Daisbach |  |
| 528822 | 5716528 | 06633025010-2 | Trendelburg / Deisel | Diemel |  |
| 451282 | 5617923 | 06532006070-1 | Dillenburg / Niederscheld | Dill |  |
| 457099 | 5604815 | 06532008050-1 | Ehringshausen | Dill |  |
| 452646 | 5608909 | 06532020010-1 | Sinn / Edingen | Dill |  |
| 446669 | 5618369 | 06532006020-1 | Dillenburg / Donsbach | Donsbach |  |
| 513522 | 5664624 | 06635003110-2 | Bad Wildungen / Wega | Eder |  |
| 510186 | 5667390 | 06635009030-1 | Edertal / Bergheim | Eder |  |
| 530545 | 5665653 | 06634003050-1 | Felsberg | Eder |  |
| 487488 | 5658826 | 06635011020-1 | Frankenberg (Eder) | Eder |  |
| 521013 | 5663854 | 06634005020-1 | Fritzlar | Eder |  |
| 532134 | 5670391 | 06634008030-1 | Guxhagen / Ellenberg | Eder |  |
| 527005 | 5652379 | 06634009060-1 | Homberg (Efze) | Efze |  |
| 532434 | 5650839 | 06634011130-1 | Knüllwald / Remsfeld | Efze |  |
| 533593 | 5645951 | 06634011170-1 | Knüllwald / Wallenstein | Efze |  |
| 523147 | 5622368 | 06535001060-1 | Alsfeld / Eifa | Eifa |  |
| 521621 | 5603332 | 06535012030-1 | Lautertal (Vogelsberg) / Eichenrod | Eisenbach |  |
| 432100 | 5597834 | 06533004030-1 | Dornburg / Langendernbach | Elbbach |  |
| 515881 | 5668142 | 06634005110-1 | Fritzlar / Züschen | Elbe |  |
| 525732 | 5667085 | 06634007060-1 | Gudensberg / Maden | Ems |  |
| 443799 | 5577001 | 06533014040-1 | Selters (Taunus) / Niederselters | Emsbach |  |
| 445710 | 5615740 | 06532004020-1 | Breitscheid / Erdbach | Erdbach |  |
| 516800 | 5623784 | 06535001120-1 | Alsfeld / Leusel | Erlenbach |  |
| 478036 | 5563293 | 06434001030-1 | Bad Homburg / Ober Erlenbach | Erlenbach |  |
| 507091 | 5616979 | 06535005020-1 | Gemünden (Felda) / Ehringshausen | Felda |  |
| 551360 | 5637425 | 06632002030-1 | Bad Hersfeld | Fulda |  |
| 536537 | 5686015 | 06611000631-1 | Kassel / Wolfsanger | Fulda |  |
| 554350 | 5641784 | 06632012080-1 | Ludwigsau / Mecklar | Fulda |  |
| 538528 | 5665955 | 06634014050-1 | Melsungen | Fulda |  |
| 543308 | 5627491 | 06632015060-1 | Niederaula | Fulda |  |
| 548434 | 5651082 | 06632018020-1 | Rotenburg a. d. Fulda / Braach | Fulda |  |
| 541962 | 5615272 | 06535015050-1 | Schlitz / Hutzdorf | Fulda |  |
| 498543 | 5535610 | 06432002010-1 | Babenhausen | Gersprenz |  |
| 490292 | 5514694 | 06437003020-1 | Brensbach | Gersprenz |  |
| 488478 | 5525794 | 06432011010-3 | Gross-Zimmern | Gersprenz |  |
|  |  |  |  |  |  |
|  |  |  |  |  |  |
|  |  |  |  |  |  |
| 520898 | 5641113 | 06634004100-1 | Frielendorf / Obergrenzebach | Grenzebach |  |
| 431194 | 5594320 | 06533004020-1 | Dornburg / Frickhofen | Grundbach |  |
| 549068 | 5625175 | 06632008070-1 | Haunetal / Neukirchen | Haune |  |
| 553035 | 5614738 | 06631015030-1 | Hünfeld | Haune |  |
| 551935 | 5608668 | 06631020030-1 | Petersberg / Marbach | Haune |  |
| 492747 | 5587202 | 06531008110-1 | Hungen / Utphe | Horloff |  |
| 498791 | 5596525 | 06531010080-3 | Laubach / Friedrichshütte | Horloff |  |
| 501218 | 5595588 | 06531010030-1 | Laubach / Gonterskirchen | Horloff |  |
| 496478 | 5595830 | 06531010080-1 | Laubach / Ruppertsburg | Horloff |  |
| 507956 | 5602612 | 06535018090-1 | Ulrichstein / Wohnfeld | Ilsbach |  |
| 492227 | 5672439 | 06635019140-2 | Vöhl / Thalitter | Itter |  |
| 503045 | 5641353 | 06634006110-1 | Gilserberg / Winterscheid | Josbach |  |
| 532202 | 5618826 | 06535006060-1 | Grebenau / Udenhausen | Jossa |  |
| 508527 | 5636305 | 06534016020-1 | Neustadt (Hessen) / Momberg | Kälbach |  |
| 535801 | 5590308 | 06631018040-1 | Neuhof / Hauswurz | Kemmete |  |
| 440509 | 5585789 | 06533013050-1 | Runkel / Hofen | Kerkerbach |  |
| 525260 | 5568467 | 06435002090-1 | Bad Soden-Salmünster / Salmünster | Kinzig |  |
| 517869 | 5563444 | 06435003060-1 | Biebergemünd / Wirtheim | Kinzig |  |
| 498507 | 5555477 | 06435007020-1 | Erlensee / Rückingen | Kinzig |  |
| 510309 | 5560931 | 06435012040-1 | Gründau / Lieblos | Kinzig |  |
| 501584 | 5556575 | 06435017000-1 | Langenselbold | Kinzig |  |
| 534949 | 5574988 | 06435025100-1 | Schlüchtern / Niederzell | Kinzig |  |
| 528375 | 5572185 | 06435028080-1 | Steinau an der Straße | Kinzig |  |
| 520932 | 5565857 | 06435029050-1 | Wächtersbach | Kinzig |  |
| 503214 | 5625363 | 06535010050-1 | Kirtorf / Lehrbach | Klein |  |
| 440162 | 5598216 | 06533011040-1 | Mengerskirchen / Waldernbach | Klingelbach |  |
| 467991 | 5637998 | 06534004010-1 | Biedenkopf | Lahn |  |
| 464587 | 5641670 | 06534004080-2 | Biedenkopf / Wallau | Lahn |  |
| 452026 | 5597968 | 06532003060-1 | Braunfels / Tiefenbach | Lahn |  |
| 474885 | 5602454 | 06531005047-1 | Giessen / Margaretenhütte/Südliche Lahnstrasse | Lahn |  |
| 468573 | 5602508 | 06532015020-1 | Lahnau / Dorlar | Lahn |  |
| 484012 | 5635499 | 06534012030-1 | Lahntal / Göttingen | Lahn |  |
| 432248 | 5583184 | 06533009080-1 | Limburg A. D. Lahn / Staffel | Lahn |  |
| 478000 | 5609673 | 06531013010-1 | Lollar | Lahn |  |
| 482706 | 5623893 | 06534014351-1 | Marburg / Cappel | Lahn |  |
| 456795 | 5599579 | 06532021020-1 | Solms / Burgsolms | Lahn |  |
| 447309 | 5591664 | 06533017110-1 | Weilburg | Lahn |  |
| 461640 | 5600602 | 06532023080-1 | Wetzlar / Steindorf | Lahn |  |
| 504276 | 5581707 | 06440016130-1 | Nidda / Schwickartshausen | Laisbach |  |
| 434918 | 5595120 | 06533016010-1 | Waldbrunn (Westerwald) / Ellar | Lasterbach |  |
| 529910 | 5610034 | 06535011040-1 | Lauterbach (Hessen) | Lauter |  |
| 457303 | 5608406 | 06532008080-1 | Ehringshausen / Kölschhausen | Lemp |  |
| 547138 | 5679507 | 06633012020-1 | Helsa | Losse |  |
| 536857 | 5600728 | 06631011030-1 | Grossenlüder / Kleinlüder | Lüder |  |
| 486283 | 5613295 | 06531001010-1 | Allendorf (Lumda) | Lumda |  |
| 495167 | 5609559 | 06531006080-1 | Grünberg / Lumda | Lumda |  |
| 488860 | 5614004 | 06531015040-1 | Rabenau / Londorf | Lumda |  |
| 490447 | 5552717 | 06435014013-1 | Hanau / Nordwest | Main |  |
| 453824 | 5538985 | 06436006010-1 | Hochheim Am Main / Hochheim | Main |  |
| 487109 | 5553069 | 06438008020-1 | Mühlheim Am Main | Main |  |
| 472806 | 5517884 | 06411000700-1 | Darmstadt / Eberstadt | Modau |  |
| 480902 | 5509928 | 06432013030-1 | Modautal / Brandau | Modau |  |
| 481765 | 5513983 | 06432013040-1 | Modautal / Ernsthofen | Modau |  |
| 477738 | 5518545 | 06432014030-1 | Mühltal / Nieder-Ramstadt | Modau |  |
| 470471 | 5516362 | 06432018040-1 | Pfungstadt | Modau |  |
| 460827 | 5517235 | 06433013000-1 | Stockstadt | Modau |  |
| 499908 | 5505151 | 06437011030-1 | Michelstadt / Steinbach | Mümling |  |
| 480469 | 5558562 | 06440003010-1 | Bad Vilbel | Nidda |  |
| 495400 | 5569341 | 06440001010-1 | Altenstadt | Nidder |  |
| 499793 | 5574554 | 06440010020-1 | Glauburg / Stockheim | Nidder |  |
| 494011 | 5526419 | 06432010080-1 | Groß-Umstadt / Richen | Ohlebach |  |
| 490277 | 5630830 | 06534011050-1 | Kirchhain / Großseelheim | Ohm |  |
| 487639 | 5632821 | 06534014341-1 | Marburg / Ginseldorf | Ohm |  |
| 511146 | 5604774 | 06535018050-1 | Ulrichstein / Ober-Seibertenrod | Ohm |  |
| 509937 | 5606246 | 06535018080-1 | Ulrichstein / Unter-Seibertenrod | Ohm |  |
| 523078 | 5565052 | 06435001000-1 | Bad Orb | Orb |  |
| 508946 | 5603599 | 06535018010-1 | Ulrichstein / Bobenhausen II | Rauchel |  |
| 451012 | 5536396 | 06433005000-1 | Ginsheim-Gustavsburg | Rhein |  |
| 525760 | 5661175 | 06634025090-1 | Wabern | Riedwiesengraben |  |
| 488557 | 5536856 | 06438012020-1 | Rödermark / Ober-Roden | Rodau |  |
| 466796 | 5624174 | 06534010100-1 | Gladenbach / Römershausen | Römershäuser Bach |  |
| 487035 | 5633769 | 06534006020-1 | Cölbe / Bürgeln | Rotes Wasser |  |
| 430794 | 5592409 | 06533004040-1 | Dornburg / Thalheim | Salzbach |  |
| 466051 | 5622806 | 06534003080-1 | Bad Endbach / Wommelshausen | Salzböde |  |
| 475257 | 5618240 | 06534013020-1 | Lohra / Damm | Salzböde |  |
| 524032 | 5597006 | 06535007080-1 | Grebenhain / Ilbeshausen-Hochwaldhausen | Schlitz |  |
| 530829 | 5598731 | 06535008060-1 | Herbstein / Schlechtenwegen | Schlitz |  |
| 532556 | 5601132 | 06535008080-1 | Herbstein / Stockhausen | Schlitz |  |
| 520135 | 5623075 | 06535001010-1 | Alsfeld | Schwalm |  |
| 521518 | 5657236 | 06634001050-1 | Borken (Hessen) / Gombeth | Schwalm |  |
| 519779 | 5634576 | 06634021020-1 | Schrecksbach / Röllshausen | Schwalm |  |
| 519719 | 5613479 | 06535017090-1 | Schwalmtal / Vadenrod | Schwalm |  |
| 529383 | 5597468 | 06535008070-1 | Herbstein / Steinfurt | Schwarza |  |
| 461045 | 5532049 | 06433009000-1 | Nauheim | Schwarzbach |  |
| 456803 | 5530188 | 06433014040-2 | Trebur | Schwarzbach |  |
| 501292 | 5570684 | 06440004070-1 | Büdingen / Düdelsheim | Seemenbach |  |
| 512805 | 5573065 | 06440004120-1 | Büdingen / Rinderbügen | Seemenbach |  |
| 500767 | 5483362 | 06437015010-1 | Sensbachtal / Hebstahl | Sensbach |  |
| 460766 | 5619123 | 06532019040-1 | Siegbach / Uebernthal | Siegbach |  |
| 542829 | 5565183 | 06435027030-1 | Sinntal / Jossa | Sinn |  |
| 458662 | 5595094 | 06532003020-1 | Braunfels / Bonbaden | Solmsbach |  |
| 557000 | 5634109 | 06632019070-1 | Schenklengsfeld / Malkomes | Solz |  |
| 473154 | 5641039 | 06534004050-1 | Biedenkopf / Engelbach | Treisbach |  |
| 504367 | 5692682 | 06635018070-1 | Twistetal / Twiste | Twiste |  |
| 508149 | 5696261 | 06635020060-1 | Volkmarsen | Twiste |  |
| 530158 | 5584106 | 06535004040-1 | Freiensteinau / Holzmühl | Ürzeller Wasser |  |
| 482269 | 5577597 | 06440002010-1 | Bad Nauheim | Usa |  |
| 471214 | 5578593 | 06434011020-1 | Usingen / Kransberg | Usa |  |
| 444230 | 5597181 | 06533012020-1 | Merenberg / Barig-Selbenhausen | Vöhlerbach |  |
| 519533 | | 5588041 | 06535007130-2 | Grebenhain / Volkartshain | Water without name |
| 520932 | | 5617362 | 06535017030-3 | Schwalmtal / Hopfgarten | Water without name |
| 479781 | | 5618717 | 06534020080-1 | Weimar / Roth | Water without name |
| 569629 | 5668372 | 06636014040-1 | Wehretal / Reichensachsen | Wehre |  |
| 455037 | 5587630 | 06533018110-1 | Weilmünster | Weil |  |
| 449650 | 5590070 | 06533019040-1 | Weinbach / Freienfels | Weil |  |
| 569849 | 5676318 | 06636003010-1 | Eschwege / Albungen | Werra |  |
| 480037 | 5492378 | 06431017020-1 | Mörlenbach | Weschnitz |  |
| 537492 | 5717642 | 06633021030-1 | Oberweser / Gieselwerder | Weser |  |
| 541880 | 5705717 | 06633022020-1 | Reinhardshagen / Veckerhagen | Weser |  |
| 479529 | 5644072 | 06534015010-1 | Münchhausen | Wetschaft |  |
| 486055 | 5595766 | 06531011060-1 | Lich | Wetter |  |
| 492418 | 5597907 | 06531011090-1 | Lich / Ober-Bessingen | Wetter |  |
| 508498 | 5634361 | 06534016030-1 | Neustadt (Hessen) | Wiera |  |
| 498002 | 5645568 | 06635012020-1 | Gemünden (Wohra) | Wohra |  |
| 488825 | 5626012 | 06534014343-1 | Marburg / Schröck | Würf |  |
| 484615 | 5618433 | 06534008040-1 | Ebsdorfergrund / Hachborn | Zwester Ohm |  |
